# First X-ray spectral ptychography and resonant ptychographic computed tomography experiments at the SWING beamline from Synchrotron SOLEIL

**DOI:** 10.1107/S1600577524003229

**Published:** 2024-05-21

**Authors:** Anico Kulow, Javier Pérez, Redhouane Boudjehem, Eric Gautier, Sébastien Pairis, Samy Ould-Chikh, Jean-Louis Hazemann, Julio César da Silva

**Affiliations:** a Univ. Grenoble Alpes, CNRS, Grenoble INP, Institut Néel, 25 Avenue des Martyrs, BP 166, 38042Grenoble, France; b Synchrotron Soleil L’Orme des Merisiers, Départementale 128 91190Saint-Aubin France; c SPINTEC, Univ. Grenoble Alpes, CEA, CNRS, 17 Rue des Martyrs, 38054Grenoble, France; d KAUST, Thuwal, Saudi Arabia; Australian Synchrotron, Australia

**Keywords:** spectral ptychography, resonant ptychography, ptychographic X-ray computed ptychography, complex refractive index

## Abstract

X-ray ptychography and ptychographic computed tomography have seen a rapid rise since the advent of fourth-generation synchrotrons with a high degree of coherent radiation. In addition to quantitative multiscale structural analysis, ptychography with spectral capabilities has been developed, allowing for spatial-localized multiscale structural and spectral information of samples. The SWING beamline of Synchrotron SOLEIL has recently developed a nanoprobe setup where the endstation’s first spectral and resonant ptychographic measurements have been successfully conducted. A metallic nickel wire sample was measured using 2D spectral ptychography in XANES mode and resonant ptychographic tomography. From the 2D spectral ptychography measurements, the spectra of the components of the sample’s complex-valued refractive index, δ and β, were extracted, integrated along the sample thickness. By performing resonance ptychographic tomography at two photon energies, 3D maps of the refractive index decrement, δ, were obtained at the Ni *K*-edge energy and another energy above the edge. These maps allowed the detection of impurities in the Ni wire. The significance of accounting for the atomic scattering factor is demonstrated in the calculation of electron density near a resonance through the use of the δ values. These results indicate that at the SWING beamline it is possible to conduct state-of-the-art spectral and resonant ptychography experiments using the nanoprobe setup.

## Abstract 2

The first X-ray spectral ptychography and resonant ptychographic computed tomography experiments at the SWING beamline at Synchrotron SOLEIL are described. To illustrate the application of the techniques, a metallic Ni wire sample is measured.

## 1. Introduction

X-ray ptychography (Rodenburg & Faulkner, 2004 ▸; Rodenburg *et al.*, 2007 ▸; da Silva & Menzel, 2015 ▸) is a well established nano microscopy technology, which is especially helpful for material characterization. It allows resolving the microstructure of samples with a spatial resolution not limited by the X-ray optics or the beam size at the sample position. In far-field X-ray ptychography, a spatially confined coherent illumination is used to scan the sample, with a partial overlap between adjacent scan positions. Relatively large regions of hundreds of µm^2^ of a sample can be scanned in a reasonable amount of time. Compared with direct imaging scanning methods, fewer scan points are necessary to reach similar spatial resolution and field of view.

Nowadays, thanks to the latest advances in computer technology and the increasing computational and storage power of modern computers and computing clusters, 2D ptychography has become a routine technique. Quasi-online retrieval of real space projection images from speckle patterns is now a well established protocol. Despite its promising potential, ptychographic X-ray computed tomography (PXCT), the 3D version of ptychography, has not yet gained widespread popularity like its 2D counterpart. This is due to the significant challenge of ensuring high resolution, which requires the setup to maintain a high level of stability. Additionally, the speed of the motors plays a crucial role during a PXCT measurement as hundreds to thousands of ptychographic scans are required. A complete PXCT experiment may take several hours despite the advanced and fast translation motors. It is crucial that the motors run reliably during this period to stabilize the sample’s position and perform accurate scans at each measurement angle. Although ptychographic reconstruction can address positioning errors and instabilities, the instrumental setup is the primary factor determining the result’s quality and resolution. Typically, a precise metrology system, *e.g.* interferometry, is used to track unavoidable vibrations and thermal drifts to ensure optimal performance (Holler *et al.*, 2012 ▸; Engblom *et al.*, 2020 ▸; Schropp *et al.*, 2020 ▸).

Despite the challenges of implementing PXCT, some synchrotron beamlines have successfully overcome them and are actively pushing forward in this field. Some examples are the cSAXS beamline at the Swiss Light Source (*e.g.* Dierolf *et al.*, 2010 ▸; da Silva *et al.*, 2015 ▸; Weber *et al.*, 2022 ▸; Holler *et al.*, 2014 ▸, 2018 ▸, 2019 ▸), the upgraded PtyNAMi setup at the beamline P06 at PETRA III (*e.g.* Schropp *et al.*, 2020 ▸), the I13 Coherence Branchline at the Diamond Light Source (*e.g.* Sala *et al.*, 2019 ▸; Batey *et al.*, 2022 ▸) and the NanoMAX beamline at the MAX IV synchrotron (*e.g.* Kahnt *et al.*, 2020 ▸).

X-ray ptychography can be extended with spectral capabilities by making use of the energy-dependent absorption and phase shift near resonances of an element (Hoppe *et al.*, 2013 ▸; Shapiro *et al.*, 2014 ▸; Hirose *et al.*, 2018 ▸). Spectral X-ray ptychography combines ptychography’s high resolution and large sample volumes with chemical sensitivity. This technique has already been reported to be used by a few synchrotron beamlines: the cSAXS beamline at SLS/PSI (Ihli *et al.*, 2018 ▸; Gao *et al.*, 2021 ▸), the BL29XUL beamline at SPring8 (Hirose *et al.*, 2018 ▸, 2019 ▸) and the P06 beamline at PETRA III at DESY (Hoppe *et al.*, 2013 ▸) are some examples. However, the combination of PXCT with spectroscopy is not yet widely used, but creating the appropriate capabilities at the beamlines will grant access to this powerful technique for a broader community.

In this context, the SWING beamline at SOLEIL joins such a select group of beamlines. Here, we present the first resonant and spectral ptychography measurements using the nano­probe setup at that beamline. The beamline is primarily dedicated to small- and wide-angle scattering (SAXS and WAXS) for studies in structural biology, physics and chemistry of soft matter. Still, the optical setup offers the right conditions for ptychography. With the addition of a specially designed nanoprobe instrument, the beamline meets the requirements for performing ptychography and PXCT. This setup is a user-friendly compact portable system that can be installed and dismounted within less than one day. We conducted experiments on an Ni wire to demonstrate the technique and its feasibility at this endstation.

The rest of this paper is divided into five sections. Section 2 provides a brief introduction to the nanoprobe setup at the SWING beamline. Section 3 proposes an overview of far-field ptychography and PXCT and a brief introduction to spectral and resonant 2D and 3D ptychography. The experiments are described in Section 4, and Section 5 presents the results obtained using photon energies around the metallic Ni *K*-edge on a nickel wire.

## 2. Nanoprobe setup at SWING

The SWING beamline is an undulator-based beamline at Synchrotron SOLEIL. Primary optics are an Si-111 monochromator and a vertically focusing mirror in its flat configuration to remove high-order harmonics. A horizontal secondary source is created 24 m downstream of the source, with a pair of slits situated just after the mirror. As the calculated coherence length is 260 µm × 90 µm (v × h), at 31.5 m distance from the primary source, a second pair of slits is used to define the coherent beam to an area of 80 µm × 80 µm, directly before the working optics, a Fresnel zone plate (FZP) with 250 µm diameter and an outer zone width of 100 nm. The photon flux delivered at the sample position (at 31.65 m) for the experiments presented here is 1.2 × 10^8^ photons s^−1^ at an energy of 8.3 keV or a wavelength, λ, of 1.494 Å.

**Figure 1:**
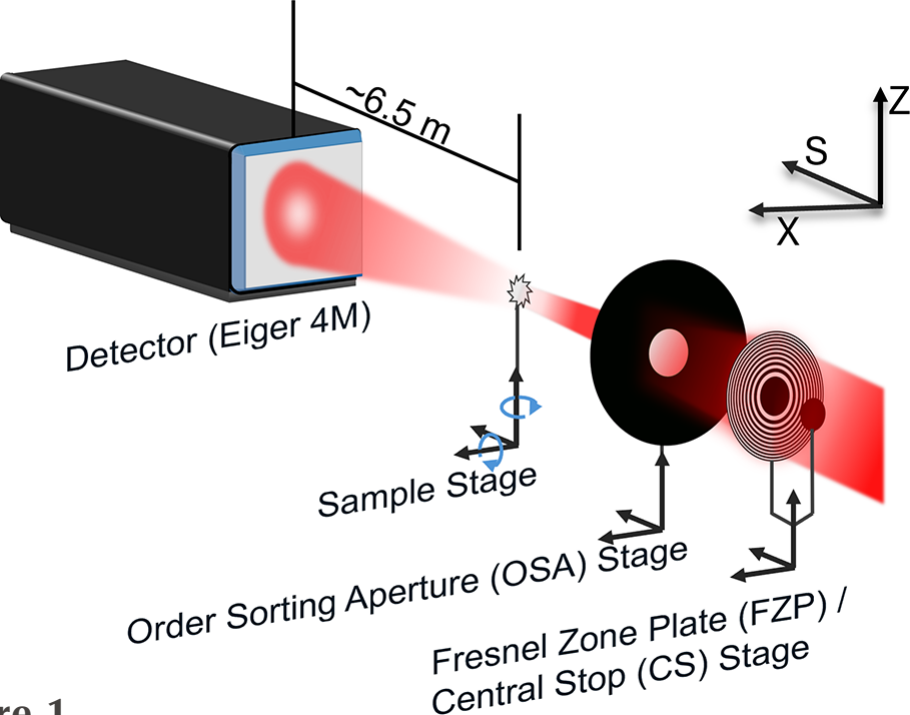
General scheme of the nanoprobe setup, introducing the coordinate system and indicating the degrees of freedom of sample-, OSA- and FZP/CS-stages. The beam path is along the *S* direction.

The general scheme for the nanoprobe setup is shown in Fig. 1 ▸. It has a one-piece aluminium stage support for high rigidity, which bears the FZP and central stop (CS) stage, the order-sorting aperture (OSA) stage, and the sample stage, as well as an interferometer support structure. A plexiglass cover hinders air circulation and helps to keep the temperature stable. The whole setup is portable, providing high flexibility at the beamline to change between different experiments. The optics consist of an FZP and a CS with a diameter of 40 µm made of 50 µm-thick Pt on one stage (at 31.5 m from the synchrotron source) with interferometric monitoring and an OSA stage (at 31.6 m from the synchrotron source), both with three linear degrees of freedom in the *S*-, *X*- and *Z*-direction. The OSA has a diameter of 40 µm and is made of 250 µm-thick Pt. The sample stage has long-range (>10 mm) *SXZ* linear drives, high-precision scanning motors in the *X*- and *Z*-directions, a short-range high-resolution *R*_*x*_ rotation, and a full-range *R*_*z*_ rotation for tomography measurements. The sample position and orientation are also monitored by interferometric heads, two in the *X*- and three in the *Z*-direction. The 2D detector, an Eiger 4M with 2068 × 2162 pixels of 75 µm × 75 µm size, is placed in a vacuum up to 6.5 m downstream of the sample. For more information about the nanoprobe setup and the control and data acquisition system, see Engblom *et al.* (2020 ▸).

## 3. Methods

### 3.1. Far-field X-ray ptychography and PXCT

In far-field X-ray ptychography, the amplitude and phase of the transmitted wave past the sample change due to the electron density inhomogeneities of the sample. The outgoing wavefield ψ(**r**; **R**) directly behind the sample can be described for each transverse scan position **R** = (*R*_*x*_, *R*_*z*_), as the product of the complex transmission function of the sample *O*(**r**) and the complex probe function *P*(**r** − **R**),
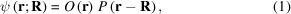
where **r** = (*r*_*x*_, *r*_*z*_) represents the transverse coordinates of the wavefunction. Since the detector is located in the far-field regime (Fraunhofer), the propagation of the outgoing wavefield can be described by a Fourier transformation so that the normalized diffraction intensity pattern at the detector can be written as
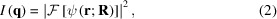
with **q** = (*q*_*x*_, *q*_*z*_) the transverse reciprocal space coordinate, where *q* = 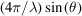 and 2θ is the scattering angle. As only intensities are acquired, the phase information of 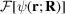 is lost but can be retrieved using iterative reconstruction algorithms (Gerchberg & Saxton, 1972 ▸; Thibault *et al.*, 2009 ▸). The algorithms used in this process aim to find functions that account for the measured intensities of the diffraction or speckle patterns. The complex-valued probe and object functions must be consistent with each ‘view’ of the patterns, and, at the same time, overlapping areas of adjacent scan regions in the object plane must agree. The probe component is assumed to be unique and constant throughout a 2D scan. This is one of the reasons why the stability of the setup is crucial. The overlapping probe positions allow for the simultaneous reconstruction of the phase and amplitude of the projected object function and refinement of the probe function.

Combining X-ray ptychography with computed tomography can extend it to three dimensions. The principle of PXCT is as follows (Dierolf *et al.*, 2010 ▸): the ptychographic scans are conducted repeatedly at various angles between 0° and 180° or 0° and 360°; after processing and aligning the ptychographic projections, computed tomography approaches are used to reconstruct the 3D volume; the result comprises quantitative 3D maps that represent the complex refractive index,
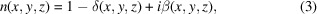
where δ relates to electronic density, while β relates to the absorption in the sample.

### 3.2. Resonant and spectral X-ray ptychography and ptychographic computed tomography

The values of δ and β for a given sample change abruptly near absorption edges but vary slowly far from them. This property can add spectral information to (2D or 3D) ptychographic datasets. Therefore, the sample is measured at different energies at and around the *K*-edge absorption energy of an element of interest. Changes in the images’ gray level measured at different energies can be attributed to the element of interest. This can be better understood by expressing the refractive index in terms of atomic scattering factors *f*_0_, *f*′ and *f*′′,
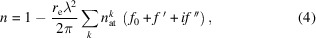
with *r*_e_ the classical electron radius, λ the photon wavelength, *n*_at_ the atomic density, and the sum going over the components of the sample voxel. Comparing equations (4) and (3), we can write
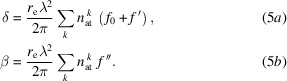
Far from an absorption edge, the changes in the atomic scattering factors *f*′ and *f*′′ with photon energy are negligible but show abrupt changes near the resonant energies, which are specific for each chemical element (Hippert *et al.*, 2005 ▸). Such modifications allow the localization of an element of interest and X-ray absorption spectroscopic information, with the large fields of view and high resolution provided by ptychography. Furthermore, the atomic density for this specific component can be calculated from a δ difference measurement when the *f*′ spectrum is known (Donnelly *et al.*, 2015 ▸),
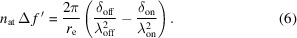
The subscripts ‘on’ and ‘off’ correspond to the different δ values measured at different energies, at the *K*-edge (on-resonant) and away from the *K*-edge (off-resonant), which correspond to different wavelengths λ.

For a 2D ptychography experiment, the results are projected images of the phase and amplitude of the sample transmission function integrated over the sample thickness. Therefore, the thickness-averaged refractive index of each pixel can only be calculated when the sample thickness is known, which is not always possible. The equation to calculate the thickness averaged δ value from the reconstructed integrated phase shift ϕ in the sample is
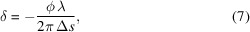
with the sample thickness Δ*s*. Such a calculation can be done for every pixel or a region in the sample where the values are averaged.

β is related to the linear absorption coefficient μ by
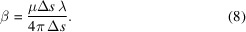
The absorption μΔ*s* is experimentally determined according to the law of Lambert–Beer, which can be written as
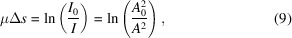
where *I* is the transmitted intensity from a pixel or averaged from a region of interest in the reconstructed sample, that is, the square of the reconstructed amplitude. It is normalized by the mean of an area outside the sample where no absorption is expected,*I*_0_. The intensities correspond to the squared amplitudes in and outside the sample, *A* and *A*_0_, respectively, which, in turn, are the real part of the direct reconstruction result.

More details about the δ and β spectra calculation can be found in the *Results*[Sec sec5] section.

## 4. Experimental

### 4.1. Sample

We measured a sample of metallic nickel prepared from a nickel wire in a truncated cone shape by focused ion beam (FIB) milling, resulting in a mean diameter of 8 µm. The sample was glued to the sample pin with ion-beam-induced deposition of Pt from an organometallic complex.

Elemental analysis by scanning electron microscopy combined with energy-dispersive X-ray spectroscopy (SEM EDX) of the original wire before FIB milling showed an Ni content of >99% and traces of Al, Si and Fe.

### 4.2. Measurements

We performed 2D spectral ptychography and 3D resonant PXCT measurements on the sample at energies around the nickel *K*-edge (8.333 keV).

The focal length of the FZP changes with energy (*e.g.* 163 mm at 8 keV and 171 mm at 8.4 keV). Hence, at higher energies the spot size on the sample is smaller. In our case, from the probe reconstruction, we found spot sizes in the sample plane of 4.7 µm × 5.8 µm (horizontal × vertical) for 8 keV, 1.9 µm × 3.9 µm for 8.3 keV and 1.05 µm × 2.66 µm for 8.4 keV. The spot size in the sample plane changes because the sample was kept at the same distance from the detector for all the energies. The rectangular form is due to the different focal length of the FZP in the horizontal and vertical directions, which, in turn, is due to the different source distances: the vertical source corresponds to the primary source, whereas the horizontal source is created by slits at 26.5 m downstream of the primary source. Additionally, the FZP is larger than the confined beam and its center is placed out of the beam axis, avoiding the central stop region.

For the 2D spectral ptychography measurement, we measured a field of view of 16 µm × 14 µm (h × v) with 242 scan positions and 100 ms exposure time per point. The scan positions are arranged in circles with a radii distance of 0.8 µm. As a rule of thumb, the scanning step size is typically chosen to be around one-third of the beam size (Bunk *et al.*, 2008 ▸), but, for consistency and considering the high number of repetitions, we chose a step size of 0.8 µm for all scans. The circles are truncated on the borders to fill the rectangular field of view.

We verified the ptychographic scan parameters to respect the sampling conditions. The condition for the real space is that the ratio *a* = *R/D* between the scanning step width *R* and the probe size *D* must be smaller than 1 to ensure overlap. At the same time, the inverse of the oversampling factor in reciprocal space, *b* = *Dp*_det_/λ*S* must also be smaller than 1, where *p*_det_ is the detector pixel size and *S* is the distance between the sample and the detector. This gives us lower and upper limits for the beam size in the sample plane for fixed scanning step width, energy, detector distance and pixel size, which are 0.8 ≤ *D* ≤ 12.9 µm for 8.3 keV and 0.8 ≤ *D* ≤ 12.8 µm for 8.4 keV. Furthermore, from these two conditions follows the oversampling ratio condition for ptychography (da Silva & Menzel, 2015 ▸), which tells us that
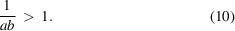
We have then checked that it is respected for the low- and high-energy configurations. We obtain the following: for the lowest measured energy of 8.3 keV the oversampling ratio is 1/(*ab*) = 16.1; for the higher energy of 8.4 keV it is 1/(*ab*) = 15.9. In both extremes, it definitely satisfies the sampling condition above.

Additionally, the FZP lens has optimal photon flux at 8 keV, and the photon flux reaching the sample decreases at higher energies. In order to verify the quality of the image reconstruction relative to the flux, we also carried out test scans at the highest energy with different values for exposure time and scanning step width, reconstructed the phase images, and checked the resolution with Fourier ring correlation (van Heel & Schatz, 2005 ▸) for two repetitions of the same scan. For the lower energies with larger spot size and higher photon flux, we used the same parameters, which tends to lead to more overlap and more photons than necessary.

Finally, the total time for each 2D ptychographic scan at a given energy was around 39 s. We repeated such a scan at 100 energies between 8.3 and 8.4 keV with an energy step of 1 eV.

For the 3D resonant ptychography measurement, the measured field of view was 14 µm × 12 µm (h × v), with 205 scan positions, arranged in circles with a distance of 0.8 µm, and 100 ms exposure time per point. We calculated the number of projected angles for an aimed resolution of 60 nm after the Crowther criterion (Crowther *et al.*, 1970 ▸),
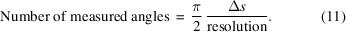
The 2D scan was repeated at 200 angles between 0° and 180°, divided into eight interlaced subtomograms with 25 angles each. The total acquisition time of the tomographic scan was around 3 h. We repeated the scan with the same measurement conditions at two energies: on-resonant at 8.337 keV and off-resonant at 8.383 keV.

## 5. Reconstructions

The ptychographic and tomographic reconstructions were performed using the SOLEIL GPU cluster Sumo with the *PtychoShelves* software package (Wakonig *et al.*, 2020 ▸). For the ptychographic reconstruction, we used 200 iterations of the difference map (DM) algorithm, followed by 100 iterations of the maximum likelihood (ML) algorithm, both implemented in the software package. After phase unwrapping, linear phase ramp removal, horizontal and vertical alignment, the tomographic reconstruction was carried out with filtered back projection (FBP) and a ram-lak filter with frequency cutoff 1.0.

## 6. Results

### 6.1. 2D spectral ptychography

**Figure 2:**
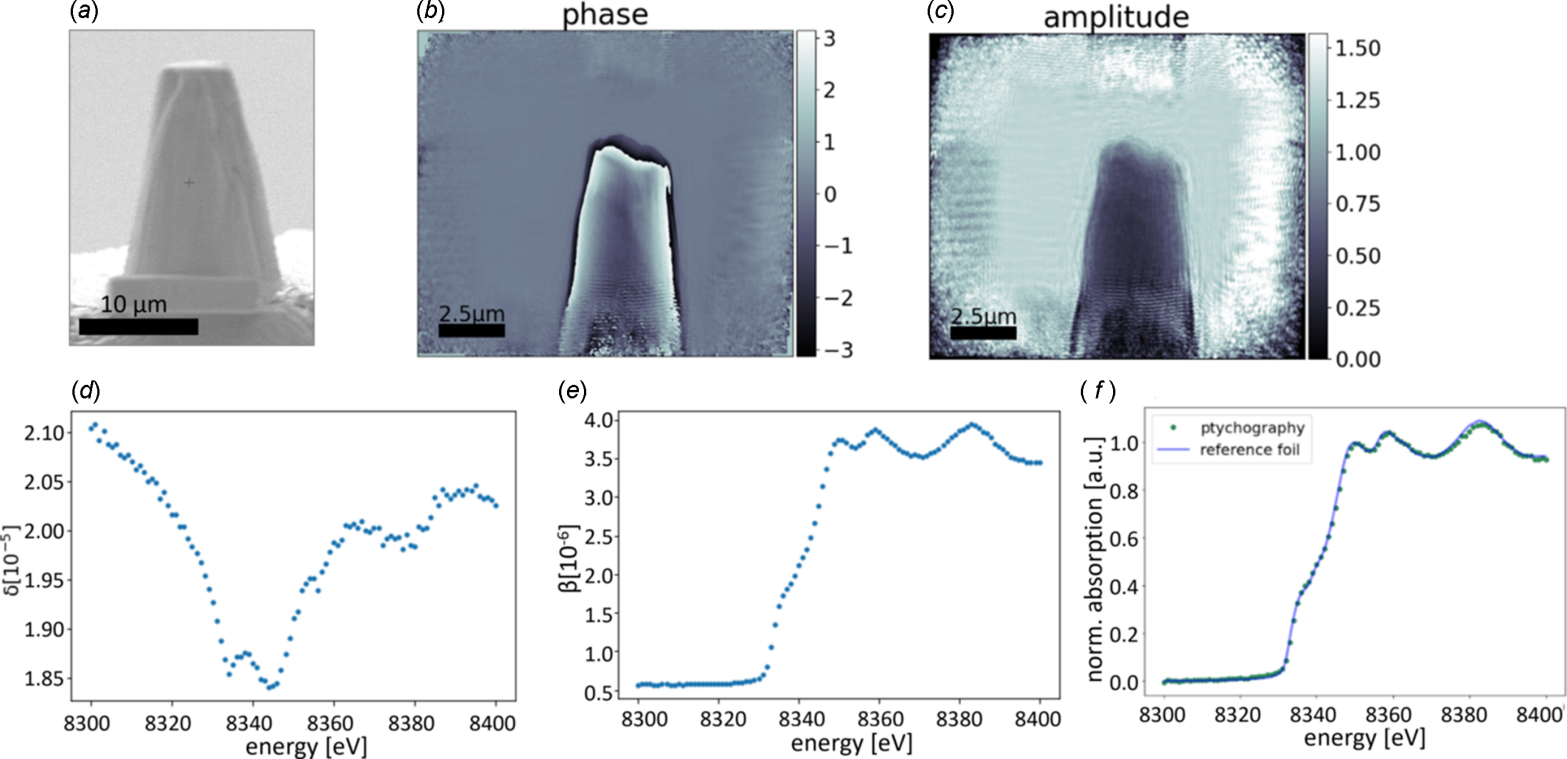
(*a*) Transmission electron microscope image of the as-prepared nickel reference sample. (*b*) Reconstructed phase. (*c*) Reconstructed amplitude. The intensity is the squared amplitude. The mean of the intensity in a region in the sample is normalized by the mean of the intensity of a region outside the sample to obtain the absorption. Bottom: the calculated δ (*d*) and β (*e*) spectra from the 2D spectral ptychography measurement. (*f*) Comparison of the normalized absorption spectrum with the normalized absorption spectrum of a classical transmission geometry XANES measurement of a nickel reference foil from the database SSHADE.

One of the reconstructions of the phase and amplitude of the nickel wire is shown in Fig. 2 ▸. Some data preparation must be done before the spectral analysis can be performed. For the data preparation and the spectral analysis, Python scripts were written using various packages, including *NumPy* (Harris *et al.*, 2020 ▸) and *Toupy* (da Silva, 2021 ▸), a collection of utils for ptychographic computed tomography.

As the pixel size of the reconstructed images depends on the energy, all reconstructions must be scaled to the same pixel size. Such an operation is performed before further processing via bi-linear interpolation with the Python *skimage.rescale* function included in the *Scikit-Image* package (Pedregosa *et al.*, 2011 ▸). Further, the phase ramp must be removed, and the phase must be unwrapped.

For the comparison of regions or single pixels, the reconstructed images recorded at different energies must be carefully aligned. For this purpose, we utilized the *skimage.registration.phase_cross_correlation* function with subpixel precision. After these preparations, δ, β and absorption spectra are extracted from the reconstructed images.

**Figure 3:**
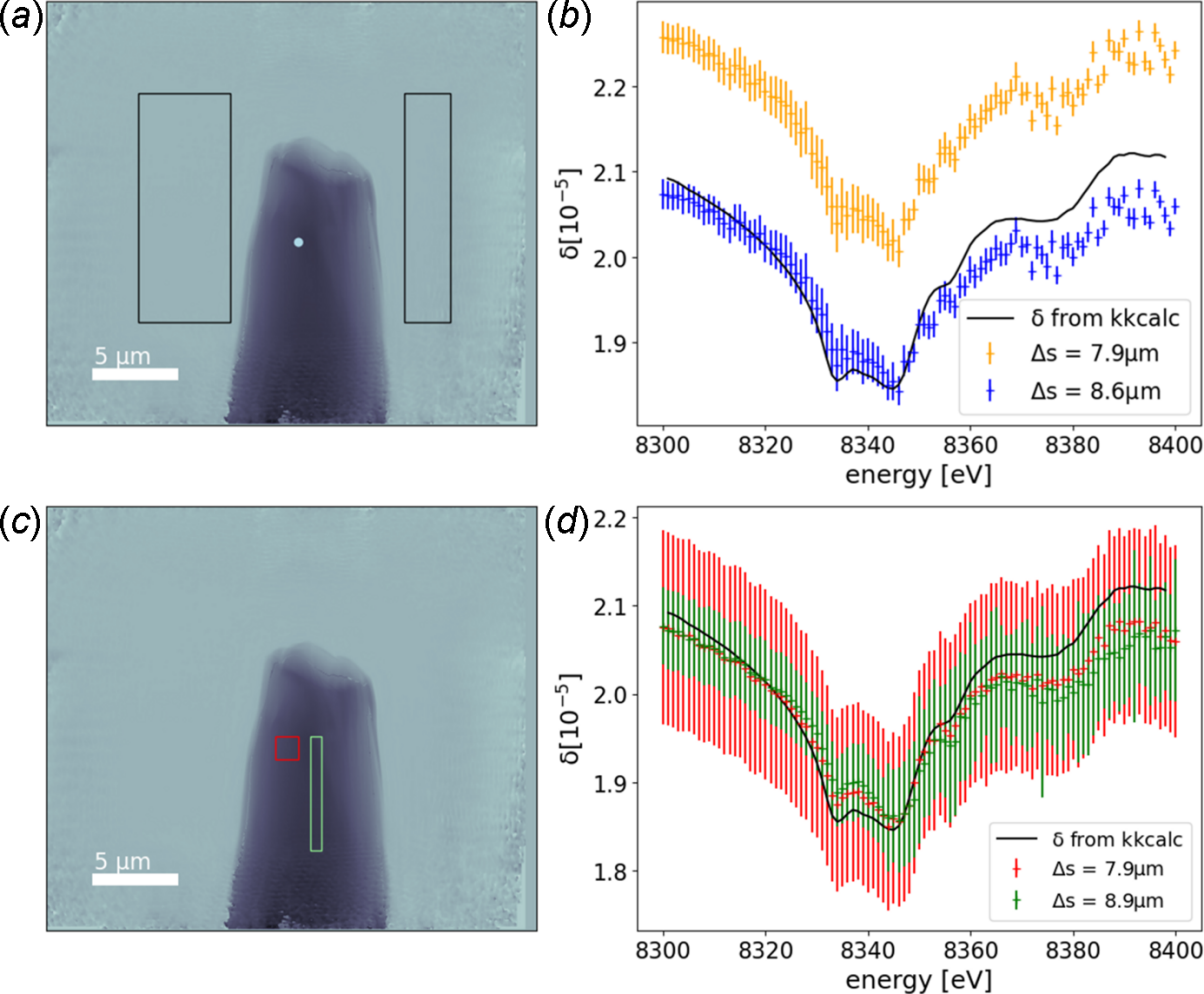
One reconstructed phase image [(*a*) and (*c*)] and the extracted δ spectra [(*b*) and (*d*)]. (*b*) δ spectrum of one pixel, indicated by the light blue dot in (*a*), with assumed thickness of Δ*s* = 8.6 µm (blue) and Δ*s* = 7.9 µm (orange), together with the δ spectrum calculated from the absorption with *kkcalc* (black line). The error bars correspond to the standard deviation of the region in air indicated by the rectangles in (*a*) where we know δ to be constant. (*d*) δ spectra averaged over two different ROIs, indicated by the rectangles in (*c*), with different assumed thicknesses. Here, the error bars correspond to the variation of calculated δ values in the ROIs, primarily due to varying thickness in the ROI itself.

The absorption is calculated using equation (9) with averaging over *I* and *I*_0_ values in the regions shown in Fig. 3 ▸ to obtain the β spectrum. This procedure is done for every energy, and the β spectrum is calculated using equation (8).

To compare the so-obtained absorption spectrum with a conventionally measured one, we used the μΔ*s* spectrum, normalized it with *Athena* software (Ravel & Newville, 2005 ▸), and compared it with a classical XANES spectrum of a nickel reference foil measured in transmission geometry (Proux, 2018 ▸; Kieffer & Testemale, 2016 ▸), and also normalized with *Athena*. The comparison of the two spectra shows an excellent agreement [see Fig. 2 ▸(*f*)].

**Figure 4:**
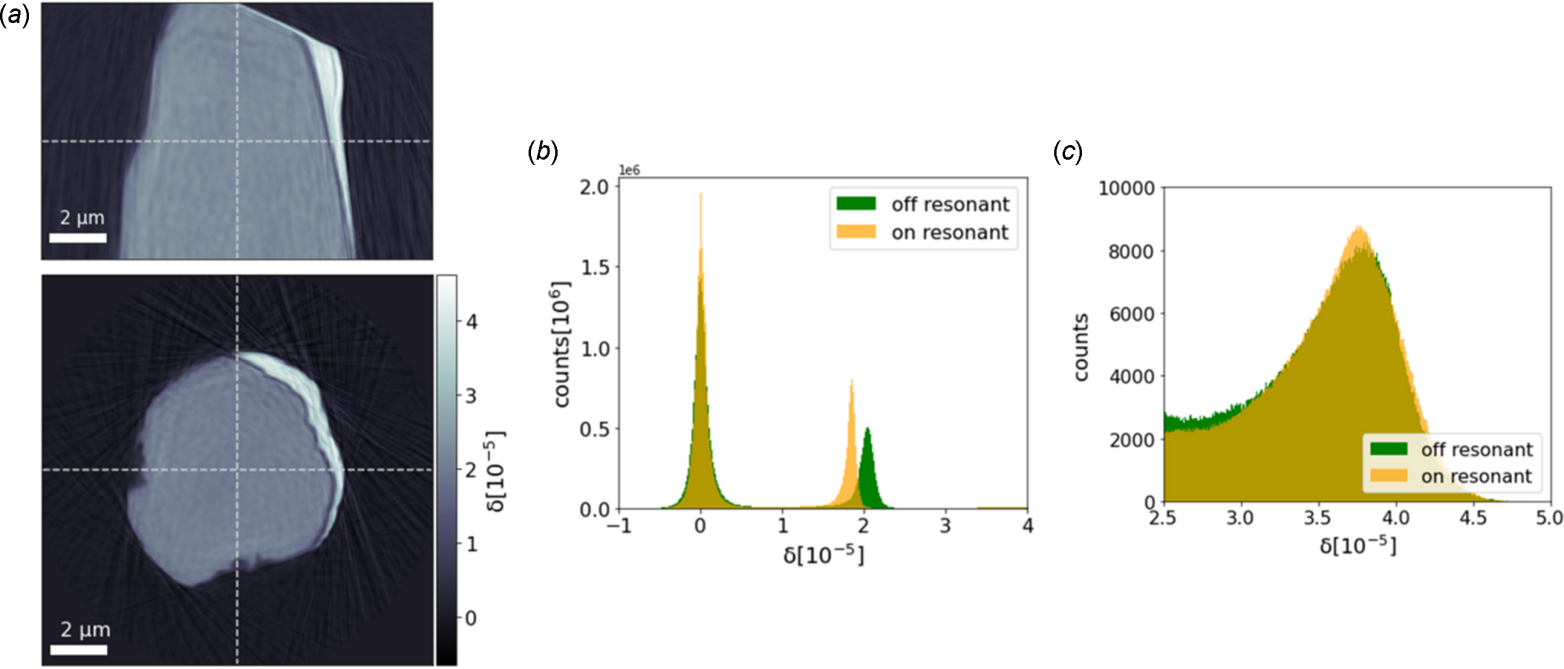
(*a*) Orthogonal cuts through the reconstructed δ volume measured at off-resonant energy. The dashed lines indicate the positions of the cuts. (*b,*c**) Histograms of the δ values of the reconstructed volume at two different energies. The peak around 0 corresponds to the air around the sample, the peak around 2 × 10^−5^, which shifts with energy, corresponds to Ni, and the peak around 3.75 × 10^−5^ [zoom in (*c*)] corresponds to a deposit on the Ni wire.

We have checked the consistency of the spectra and the thickness assumption by use of the Kramers–Kronig relation (Kronig, 1926 ▸; Kramers, 1927 ▸), which describes the correlation between the real and imaginary part of the atomic scattering factor, and which is implemented for example, in the program *kkcalc* (Watts, 2014 ▸). Here, for calculating the real part (*f*′ or δ) from the imaginary part (*f*′′ or β), the data are first automatically scaled to the tabulated values before the transformation is performed. That is, a normalization is performed. As a result, all β spectra that are calculated with different assumed thicknesses [a variable in equation (9)] give the same δ spectrum when processed by *kkcalc*. As the calculated δ spectrum depends on the assumed thickness, it can be vertically shifted by changing the thickness value. As shown in Fig. 3 ▸, the best match between the δ spectrum calculated directly from the experimental data and the one calculated by *kkcalc* is obtained for different assumed thicknesses depending on the considered pixel or region of interest (ROI). We consider as uncertainty of one single pixel the standard deviation of a region in air, where we know the δ values to be constant. It changes slightly with energy and is between 0.01 × 10^−5^ and 0.035 × 10^−5^. The error bars for the different ROIs (see Fig. 3 ▸) correspond to the standard deviation of the single pixel values that were used to calculate the mean δ. We chose one thickness value for each ROI, so that important deviations are expected as the thickness is not uniform inside one ROI. Besides the problem with the varying thickness, the comparison of the so-found thickness values with the tomogram shows that the diameter of the wire is over-estimated in 2D calculations. This can be explained by the fact that we neglected the higher electron density deposit (see Section 6.2 and Fig. 4 ▸). This deposit is at the origin of a more significant phase shift than a pure Ni sample could explain. In turn, we would expect a pure Ni wire of a larger diameter than the sample for the same phase shift.

### 6.2. 3D resonant ptychography

The result of the tomographic reconstruction is a 3D map of the β and δ values of the reconstructed volume. The voxel size is (26.6 nm)^3^ for the off-resonant tomograms and (26.7 nm)^3^ for the on-resonant tomograms. The resolution, as found by Fourier shell correlation (FSC), is ∼60 nm, which is in the expected range for the resolution for a tomographic measurement of a sample of this thickness with 200 angles as calculated based on Crowther’s criterion.

Fig. 4 ▸ shows two orthogonal cuts through the reconstructed δ volume of the Ni wire measured at off-resonant energy and δ histograms of the reconstructed volume for both measured energies. The sample is clearly visible, as well as a deposit on one side of its surface, which comes from the sample preparation.

### 6.3. δ histograms

For further analysis, we work primarily with the δ tomograms, as the δ values are in the range of up to 4 × 10^−5^, which is about one order of magnitude larger than the β values, and the corresponding tomograms have a better signal-to-noise ratio. In the δ histograms of the on- and off-resonant measurement, three peaks are discernible in each histogram: one peak around 0, which corresponds to the air around the sample. The air peaks at on- and off-resonant energy are both centered at 0. The standard deviation of the peaks is around 0.075 × 10^−5^, corresponding to the uncertainty of the δ values for this experiment. Another peak is visible at δ values around 2 × 10^−5^, corresponding to the sample’s Ni. In the on-resonant case the peak is centered at 1.85 × 10^−5^, while in the off-resonant case it is centered at 2.03 × 10^−5^. This difference is the base of resonant ptychography, as explained above. A third peak corresponds to a deposit on the sample and is located at around 3.75 × 10^−5^. This value could correspond to a mixture of Pt from the FIB deposition of the sample on the pin and Ga from the Ga FIB instrument. Reference values from the Chantler database (Chantler, 2000 ▸) are summarized in Table 1 ▸.

**Figure 5:**
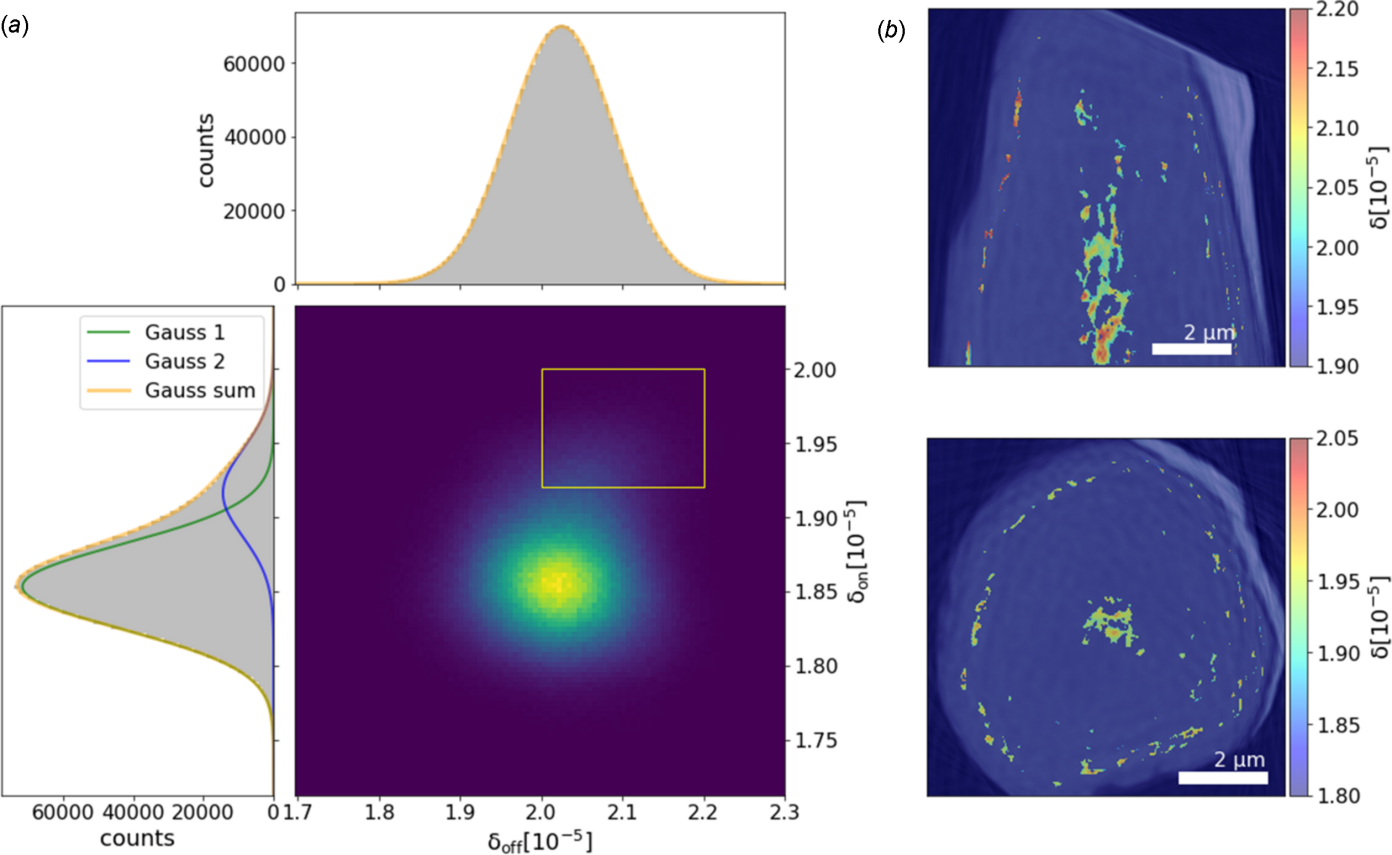
(*a*) Bivariate histogram of the δ_off_ and δ_on_ values of a region in the center of the Ni wire and projected histograms. While the δ_off_ histogram has a Gaussian shape, the δ_on_ histogram is better described by combining two Gaussian curves. This behavior indicates the contamination of the Ni wire with a material that has a similar δ_off_ but a different δ_on_ value. The voxels with this combination of δ values are indicated by the rectangle in the bivariate histogram. The localization of these voxels in the sample volume is shown in (*b*).

For further investigations, a region of 120 × 120 × 130 voxels in the center of the Ni wire was chosen to exclude all possible phenomena not only related to the wire material but also to sample preparation and contact with the environment. Fig. 5 ▸ shows the δ histograms of this volume for the on- and the off-resonant energy with fitted Gauss curves. The off-resonant histogram has a Gaussian shape, whereas the on-resonant histogram is better fitted with two Gaussian curves. The different shapes of the histograms can be explained by impurities of a material that has a similar δ value as Ni at *E* = 8.383 keV but not at *E* = 8.337 keV. Also, evidence for this contamination can be found in the bivariate histogram of the δ on and off values, shown in Fig. 5 ▸(*a*). In addition to the main spot, corresponding to Ni, a faint spur is discernible at higher δ on values, which can be fitted with a second Gaussian, indicating the impurities in the Ni wire.

### 6.4. Impurities in the Ni wire

We assume that these impurities contain Fe, as this element has been found in traces in the preliminary analysis. The tabulated δ value of Fe is very close to the one of Ni at 8.383 keV, and clearly different at 8.337 keV (see Table 1 ▸).

The fact that the δ values of the minor Gaussian peak attributed to Fe in the on-resonant case are significantly lower than the reference value for Fe can be explained by the partial volume effect, which means that at the given resolution no voxel contains only Fe atoms but always a mixture of Ni and Fe. We built a mask to locate the voxels in the whole volume, which have the specific combination of δ values that indicate contamination, indicated by the yellow rectangle in Fig. 5 ▸. These voxels are located mainly in the center of the wire and a circular region near the surface (see Fig. 5 ▸). To get an idea of the order of magnitude of the Fe content in these voxels, we consider the mean of the second Gaussian curve in the on-resonant δ histogram (1.92 × 10^−5^) and perform a linear combination of the Ni and Fe δ values at this energy. The result indicates around 80% of Ni in the voxels with this specific δ value. Taking the mean of all voxels in the considered region gives a δ value of 1.86 × 10^−5^, which, in turn, corresponds to a mean Fe content of around 4%.

These values should be viewed with caution, considering the uncertainty of the δ values of 0.75 × 10^−6^. Still, they can give an idea of the possibility of the method to detect different elements in a sample – that would not have been seen if measurements had been made only at off-resonant energy.

### 6.5. Electron density from δ values in the vicinity of an absorption edge

From the mean and the standard deviation of the Gaussian curves fitted to the δ histograms at on- and off-resonant energies, the following Ni δ values are obtained,
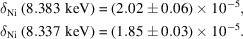
The equation to calculate the electron density from δ in the forward scattering direction is
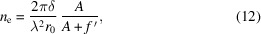
with *A* being the element’s atomic number; for Ni *A* = 28. The second term in the product approximates 1 far from the resonance energy, as *f*′ goes to 0. In the vicinity of an absorption edge, the *f*′ term cannot be neglected and accounts for the resonance effect.

With the *f*′ values from the 2D ptychographic measurement of the Ni wire, the electron density values are calculated using equation (12) from the δ values from the 3D δ tomograms,
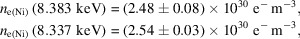
which is slightly below the literature value of pure Ni of 2.56 × 10^30^ e^−^ m^−3^. Both values are compatible with each other.

With the measurements at different energies, it is possible to cross-validate the obtained results and to show that a quantitative determination of the electron density is also possible in the vicinity of an absorption edge. However, to obtain more accurate results for the electron density, it is indispensable to know the exact values of *f*′ from a different experiment.

### 6.6. Atomic density

**Figure 6:**
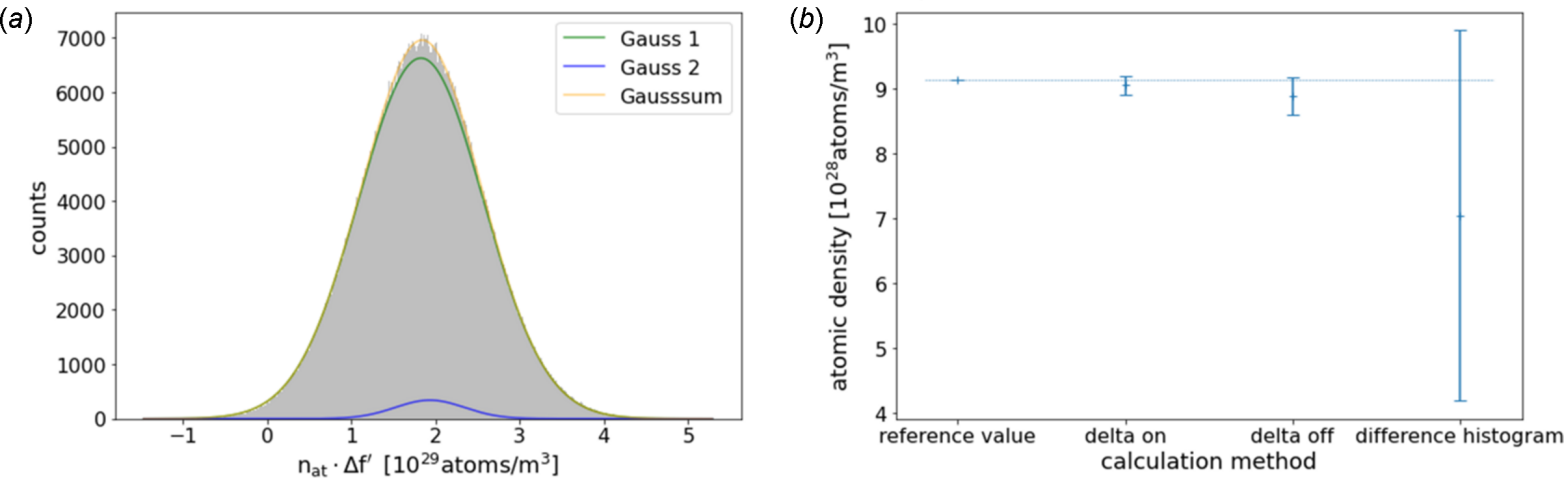
(*a*) Histogram of a region in the middle of the Ni wire of the difference tomogram, calculated from the delta tomograms [equation (6)]. From this histogram, the atomic density of Ni can be calculated by dividing by the difference of the *f*′ values at the different energies. (*b*) Comparison of atomic densities of Ni, calculated from the delta tomograms at different energies and from the difference tomogram; the horizontal line indicates the reference value.

The atomic density of Ni in the sample can be calculated from the estimated electron densities for the on- and the off-resonant δ measurements (see above) by simply dividing them by the atomic number of Ni, 28. The obtained values are
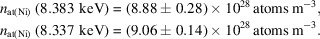
As indicated in Section 3, another possibility is to make use of equation (6) and calculate the atomic density from the *difference* tomogram. To this end, the δ-tomograms are divided by the square of the corresponding wavelength, and the difference of both is then multiplied by the pre-factor of constants [see equation (6)], yielding a tomogram of *n*_at_Δ*f*′ values. The histogram of a region in the middle of the Ni wire can be fitted with the sum of two Gaussian curves (see Fig. 6 ▸), again indicating the contribution of the impurities in the sample, giving a mean value and a standard deviation that can be used to calculate the atomic density and its uncertainty, using the *f*′ values from the 2D ptychographic measurement of the Ni wire.

The so-calculated atomic density is
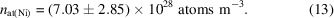
The absolute value for the atomic density is below the ones calculated from the δ_off_ and δ_on_ histograms. This reflects the presence of a considerable number of voxels with a smaller difference between δ_off_ and δ_on_ than expected for pure Ni. This indicates again impurities in the Ni wire. In addition, this could indicate inaccuracies of the *f*′-values, which influence the atomic density calculated from the difference much more than the direct calculation from the directly measured δ tomograms. The uncertainty in this way to determine the density is very high, even without considering uncertainties of the *f*′ values. It comes exclusively from the width of the difference histogram. The subtraction of the δ values, taking into account the pre-factors, results in values in the same order of magnitude as the uncertainty calculated by error propagation. This fact makes the atomic density determination by the difference method far less appropriate.

All values are compatible with but lower than the tabulated value of 9.14 × 10^28^ atoms m^−3^, a result that is in line with the slightly lower electron density.

## 7. Conclusion

This work showed the first spectral ptychography and resonant ptychographic computed tomography experiments at the SWING beamline carried out on an Ni wire as an example sample.

We could extract phase- and absorption spectra from the 2D ptychographic measurement of the sample at different energies. The absorption spectrum matches well an absorption spectrum of an Ni reference foil measured in a classical XAS experiment in transmission geometry. Such spectra can be extracted for different regions in the sample; the signal-to-noise ratio limits the spatial resolution. However, the sample is not very well suited for this type of experiment, as it has a non-uniform thickness. The spectra could have been corrected using the 3D reconstructions of the Ni wire. This was not done because we wanted to show the results of the two different experiments independently, and we expected no further valuable information. Nevertheless, our experiment showed the feasibility of 2D spectral ptychography at the SWING beamline.

Ptychographic X-ray computed tomography measurements were carried out at different energies, close to the resonance energy of Ni, at 8.337 keV, and well above the Ni *K*-edge, at 8.383 keV. From the distribution of the extracted δ values, we can deduce that there must be impurities in the Ni wire. With a mask calculated based on the combination of δ values at the different energies, we can visualize the localization of these impurities. The localization of elements in the microstructure of a sample is a great benefit of the difference measurement.

Each δ tomogram is also the base for calculating the electron density of Ni in the sample. This calculation depends on the knowledge of *f*′ at the considered energy. Using the *f*′ values extracted from the 2D spectral ptychography experiment, we obtained very reasonable electron densities from the δ tomograms acquired at different energies.

The atomic density was calculated from the electron densities at each of the two energies and also from the difference tomogram. We obtained reasonable results with both methods, but the uncertainty of the different ways is substantially different. In particular, we see a large uncertainty and important influence of the not directly measured *f*′ values for the calculation from the difference tomogram. To obtain better results with this method, more work must be put into the precise determination of the *f*′ values. However, this is made difficult by the proximity to the resonance.

Resonant and spectral ptychography can help to locate one element of interest in a sample and to extract spatially resolved chemical information. This information can be combined with the possibility of high-resolution analysis of the microstructure of micrometre-sized regions of a sample offered by ptychography. The SWING beamline provides the capability to conduct such measurements.

## Supplementary Material

Resonant ptychographic X-ray computed tomography dataset of nickel pillar from Synchrotron Soleil: https://doi.org/10.5281/zenodo.10265626

2D spectral ptychography dataset of nickel pillar from Synchrotron Soleil: https://doi.org/10.5281/zenodo.10000776

## Figures and Tables

**Table 1:** Tabulated values (Chantler database) of δ for different elements at different energies

	δ (8.337 keV)	δ (8.383 keV)
Ni	1.86 × 10^−5^	2.08 × 10^−5^
Fe	2.11 × 10^−5^	2.09 × 10^−5^
Pt	4.80 × 10^−5^	4.75 × 10^−5^
Ga	1.50 × 10^−5^	1.48 × 10^−5^
